# Glutamate transporters are involved in direct inhibitory synaptic transmission in the vertebrate retina

**DOI:** 10.1098/rsob.240140

**Published:** 2024-07-31

**Authors:** Stephanie Niklaus, Stella M. K. Glasauer, Peter Kovermann, Kulsum F. Farshori, Lucia Cadetti, Simon Früh, Nicolas N. Rieser, Matthias Gesemann, Jingjing Zang, Christoph Fahlke, Stephan C. F. Neuhauss

**Affiliations:** ^1^ Department of Molecular Life Sciences, University of Zurich, Winterthurerstrasse 190, 8057 Zurich, Switzerland; ^2^ Institute of Biological Information Processing, Molekular- und Zellphysiologie (IBI-1), Forschungszentrum Jülich, Leo-Brandt-Strasse, 52425 Jülich, Germany

**Keywords:** glutamate, transporters, inhibitory, synaptic, transmission, vertebrates

## Abstract

In the central nervous system of vertebrates, glutamate serves as the primary excitatory neurotransmitter. However, in the retina, glutamate released from photoreceptors causes hyperpolarization in post-synaptic ON-bipolar cells through a glutamate-gated chloride current, which seems paradoxical. Our research reveals that this current is modulated by two excitatory glutamate transporters, EAAT5b and EAAT7. In the zebrafish retina, these transporters are located at the dendritic tips of ON-bipolar cells and interact with all four types of cone photoreceptors. The absence of these transporters leads to a decrease in ON-bipolar cell responses, with *eaat5b* mutants being less severely affected than *eaat5b*/*eaat7* double mutants, which also exhibit altered response kinetics. Biophysical investigations establish that EAAT7 is an active glutamate transporter with a predominant anion conductance. Our study is the first to demonstrate the direct involvement of post-synaptic glutamate transporters in inhibitory direct synaptic transmission at a central nervous system synapse.

## 1. Introduction

The first visual synapse encompasses connections between pre-synaptic photoreceptors and post-synaptic bipolar and horizontal cells. Bipolar cells vertically relay the signal they receive from photoreceptors to ganglion cells, which constitute the retinal output neurons. In the photoreceptor synapse, the light-induced signal is transmitted from photoreceptors into two parallel pathways, the ON and OFF pathways mediated by two types of bipolar cells, the ON- and OFF-bipolar cells, respectively. While ON-bipolar cells depolarize upon light increments, OFF-bipolar cells depolarize to light decrements. This separation is based on the differential expression of glutamate receptors.

OFF-bipolar cells are depolarized by glutamate through AMPA/kainite receptors. Photoreceptors tonically release glutamate in darkness, which binds to the class III metabotropic glutamate receptor mGluR6 on ON-bipolar cell dendrites. This in turn causes the activation of a G-protein signalling cascade leading to the closure of the constitutively open cation conducting ion channel TRPM1 [1–3]. In incremental light, glutamate release by photoreceptors is reduced, rendering the mGluR6 signalling cascade inactive. The consequential opening of TRPM1 causes ON-bipolar cells to depolarize. This mGluR6–TRPM1 signalling cascade is evolutionarily conserved in vertebrates [2,4].

The existence of an alternative glutamatergic input mechanism for ON-bipolar cell activation was first proposed in teleost fishes and is thought to extend to vertebrates, including mammals. The initial experimental evidence for this mechanism emerged in 1979 when Saito *et al*. showed a resistance increase in carp ON-bipolar cells at photopic conditions and a decrease thereof at scotopic conditions [5]. A series of studies on different teleost retinas later demonstrated the presence of a glutamate-activated chloride current in ON-bipolar cells [6–11]. The sensitivity of this current to the non-specific glutamate transporter blocker DL-threo-beta-benzyloxyaspartate (TBOA) suggested the current to be mediated by excitatory amino acid transporters (EAATs) [9–11].

EAATs are glutamate transporters that belong to the solute carrier 1 (SLC1) family, which includes both pre- and post-synaptically expressed transporters on neurons and glia cells [12–14]. These transporters are pre- and post-synaptically expressed on neurons and glia cells. The transport of glutamate from the synaptic cleft into the cell by EAATs is electrogenic. The transmembrane concentration gradient of the co-transported Na^+^ is the transporters’ driving force with one molecule of glutamate being co-transported with 3 Na^+^ and 1 H^+^ followed by a counter-transport of 1 K^+^ (reviewed by Alleva *et al.* [15]). Interestingly, substrate binding generates a thermodynamically uncoupled, Cl^−^ conductance through EAATs with current amplitudes highly varying between different EAAT isoforms [16–20]. In essence, EAATs are bifunctional, serving as transporters, as well as anion channels, and thus are capable of modulating signal transmission by uptake of glutamate from the synaptic cleft and modulating the cell membrane potential.

EAAT5, owing to its biophysical characteristics, has been proposed to mediate the ON-response [11]. Both human and mouse EAAT5 have been extensively characterized in isolated expression systems. EAAT5 is a low-capacity transporter with a transporter current below the resolution threshold, yet it exhibits a large voltage-dependent anion conductance comparable to a bona fide Cl^−^ channel, which functions optimally at negative potentials (generated by the co- and counter-transported ions) below resolution threshold [19,21,22].

Teleosts retain the complete ancestral vertebrate complement of seven EAAT genes. Moreover, zebrafish (*Danio rerio*) EAAT1, 2, 5 and 6 even have two ohnologs stemming from an ancient whole genome duplication [23], while mammalian genomes contain five EAAT genes.

Here, we analysed the expression patterns of the two ON-bipolar cell glutamate transporters EAAT5b and EAAT7 in the zebrafish retina. We show that both transporters are co-expressed on ON-bipolar cell dendritic tips contacting all cones. Functional analysis on knockout (KO) animals showed a reduced *b*-wave response in the electroretinogram (ERG) of *eaat5b* mutants, which is larger in *eaat5b/eaat7* double mutants. Intriguingly, the implicit time (time to peak of the *b*-wave) is shorter in *eaat5b* single and *eaat5b/eaat7* mutants as compared with wild-type (WT). We analysed EAAT7 function using heterologous expression and whole-cell patch clamp recordings and demonstrated that EAAT7 is a low-capacity glutamate transporter with predominant anion conductance.

Hence, our study provides evidence of EAAT5b and EAAT7 generating hyperpolarizing currents in darkness with different kinetics. This is the first report directly showing that glutamate-mediated EAAT hyperpolarizing currents are involved in direct synaptic transmission in a central nervous synapse.

## 2. Material and methods

### 2.1. Zebrafish husbandry

Zebrafish (*Danio rerio*) of the WT strains Wik and Tübingen were kept and bred under standard conditions at 28°C in a 14/10 h light/dark cycle as described before [24]. Embryos and larvae were raised until 5 days post-fertilization (dpf) in E3 medium containing methylene blue or in 1-phenyl-2-thiourea (PTU, Sigma-Aldrich) if prevention of pigment formation was required. From 5 dpf on, larvae were raised in facility water.

The following transgenic lines were used for immunohistochemistry: *Tg(zfSWS1−5.5A:EGFP)* [25], *Tg(zfSWS2−3.5A:EGFP)* [26], *Tg(zfRh2−2:EGFP)* [27], *Tg(zfLWS:EGFP)* [28] and *Tg(zfRh1−3:EGFP)* [29], expressing EGFP in UV, blue, green, red cones and rods, respectively.

2.2. Generation of CRISPR/Cas9 KO lines

CRISPR targets were selected using the target site prediction tools www.zifit.partners.org and https://chopchop.rc.fas.harvard.edu. The following genomic target sites (GGN18) were chosen: 5′-GGTGGTGGTGGGAATCGTCA-3′ on exon 3 of *eaat5b* and 5′-GGGAACCCAAAACTCAGGTC-3′ on exon 3 of *eaat7*. The sgRNAs were prepared using a PCR-based protocol as described by Gagnon *et al.* [30] with minor adaptations. Briefly, double-strand DNA template oligos were synthesized using a Phusion High-Fidelity DNA Polymerase (New England BioLabs) with the T7 specific forward primer 5′-GAAATTAATACGACTCACTATA**GG** **N18**GTTTTAGAGCTAGAAATAGC-3′ together with the common reverse primer 5′-AAAAGCACCGACTCGGTGCCACTTTTTCAAGTTGATAACGGACTAGCCTTATTTTAACTTGCTATTTCTAGCTCTAAAAC-3′. The generated amplicon was purified after gel electrophoresis using the QIAquick Gel Extraction Kit (Qiagen) and served as template for *in vitro* transcription with MEGAshortscript T7 Transcription Kit (Ambion). Purification of synthesized sgRNA was carried out using the Megaclear Kit (Ambion) which was followed by an ethanol precipitation.

The CRISPR injection mix containing 814 ng µl^−1^ GFP-tagged Cas9 protein (kindly provided by Prof. Dr C. Mosimann and Prof. Dr M. Jinek), 150 ng µl^−1^ sgRNA and 300 mM KCl [31] was incubated at 37°C for 10 min prior injection allowing protein–sgRNA complex formation. One nanolitre of the mix was injected into the cell of a one-cell stage embryo using a FemtoJet Microinjector (Eppendorf).

Mosaic F0 founder fish were outcrossed to WT fish and resulting heterozygous F1 generation was genotyped (PCR amplification of lysed fin biopsies, cloning and sequencing of plasmids) using the following primers: 5′-TTGATGTCAGGTTTGGCG-3′ and 5′-TGATGGGTTTTCCGCTGT-3′ for *eaat5b* and 5′-ATGTCCACCACAGTAATCG-3′ and 5′-TTGAAAACAGGCCTGGAC-3′ for *eaat7*. Fish carrying a+79 base pairs (bp) indel in *eaat5b* and a −74 bp deletion in *eaat7* were used for all experiments. The *eaat5b* and *eaat7* mutant fish were genotyped by PCR amplification of the genomic region using the primers listed above. Amplicon length revealed the genotype of *eaat5b*, with a WT amplicon of 180 bp and a mutant fragment of 259 bp. The *eaat7* PCR amplicon was digested using DdeI resulting in two WT fragments of 116 and 73 bp and a sole mutant fragment of 182 bp. The *eaat5b*/*eaat7* double KO fish were generated by crossing *eaat5b* ^−/−^ to *eaat7* ^−/−^ fish.

### 2.3. Cloning of *eaat* genes and mRNA *in situ* hybridization

Fragments of *eaat* genes were PCR amplified using a JumpStart Taq polymerase (Sigma-Aldrich) and the primer pairs *eaat5b*_135_s 5′-GGAGCAGGAAGTCAAGTA-3′ with *eaat5b*_600_as 5′-GTCCGTCCCATTATCGTC-3′ and *eaat7*_142_s 5′-GTAATAGCAGGCACAGTGATG-3′ with *eaat7*_1001_as 5′-CCCAGAGCAGTGATCCAAG-3′. *In situ* probes were prepared according to the protocol described by Niklaus *et al.* [32]. *In situ* hybridization was performed on 5 dpf PTU-treated whole-mount larvae and sections of adult retinae. Adult eyes and whole larvae were fixed with 4% paraformaldehyde (PFA) overnight at 4°C. Eyes were cryoprotected in 30% sucrose (phosphate buffered saline (PBS)) overnight at 4°C, embedded in Tissue-Tek O.C.T. Compound (Sakura Finetek) and sectioned at 16 μm. Both whole mount and slide *in situ* hybridization were carried out based on the protocol published by Thisse & Thisse [33] with adaptations described in Huang *et al.* [4]. Hybridization and stringency washes were carried out at 64°C for both genes.

### 2.4. Generation of antibodies

Zebrafish peptide-specific antibodies were generated in an 87 days classical programme by Eurogentec SA (Seraing, Belgium). Guinea pigs and rabbits were immunized with the EAAT5b epitope N-PDRKKPPVPPRHLKHRDKDHCA-C and EAAT7 epitope N-KLRSGQVSSAPRNQEV-C, respectively. Guinea pig anti-EAAT5b and rabbit anti-EAAT7 antibodies were column purified by Eurogentec.

### 2.5. Immunohistochemistry

Eyes of adult fish were fixed with 4% PFA for 30 min at room temperature (RT) or with 2% trichloroacetic acid for 25 min at RT. Fixed eyes were washed twice with PBS prior to dehydration in 30% sucrose (PBS) overnight at 4°C. Eyes were embedded in Richard-Allan Scientific Neg-5 Frozen Section Medium (Thermo Fisher Scientific) and sectioned at 16 μm. Sections mounted on superfrost-coated slides (Thermo Fisher Scientific) were dried for 30 min at 37°C before staining. Sections were washed with PBS and blocked for 45 min with blocking solution (10% normal goat serum, 1% bovine serum albumin, 0.3% Triton in PBS, pH 7.4). Primary antibodies diluted in blocking solution were applied ON at 4°C. The following antibodies were used: guinea pig anti-EAAT5b (1:100), rabbit anti-EAAT7 (1:400), rabbit anti-mGluR6b (1:750) [4], mouse anti-PKCα MC5 (Novus Biologicals, NB200-586) (1:500), mouse anti-Synaptic Vesicle 2 (SV2) (DSHB, Iowa City, IA, USA) (1:100), chicken anti-GFP (A20162; Invitrogen) (1:500) and rabbit anti-PKCβC16 (E1313, Santa Cruz Biotechnology) (1:500). Primary antibodies were detected with the following secondary antibodies (all 1:500 in PBS) for 1.5 h at RT: goat anti-rabbit, goat anti-guinea pig, goat anti-chicken and goat anti-mouse all conjugated to Alexa Fluor (AF) 488, 568 or 647 (Invitrogen, Molecular Probes). For stimulated emission depletion (STED) microscopy, goat anti-guinea pig AF 488 and goat anti-rabbit Atto 594 secondary antibodies were used. To counterstain green fluorescence, Bodipy TR methyl ester (Thermo Fisher Scientific) diluted 1:300 in PDT (0.1% Triton-X100 and 1% DMSO in PBS) was applied for 20 min after washing secondary antibodies. Confocal laser scanning imaging was done with a TCS LSI confocal microscope (Leica) and STED images were taken with a CLSM-Leica SP8 inverse STED 3× microscope. The same confocal settings were used for imaging corresponding staining on mutant and WT animals.

### 2.6. Retinal histology

Prior to embedding in Technovit 7100 (Kulzer Histotechnik) 5 dpf larvae and adult eyes were fixed in 4% PFA overnight at 4°C. Embedding, sectioning and staining of histological sections were done according to the protocol described by Niklaus *et al.* [32]. Images were taken in the bright field mode of a BX61 microscope (Olympus).

### 2.7. White light and flicker electroretinography

White light ERGs were recorded on 5 dpf WT and mutant eyes in a double-blinded manner, as described in Sirisi *et al.* [34]. Briefly, larvae were dark adapted for at least 30 min and preparations prior to recordings, including eye dissection, positioning eye and recording pipette, were done under a dim red light to prevent bleaching of photopigment. The larval eye was removed and placed on a filter paper on top of an agarose gel. The reference electrode was inserted into the agarose gel and the recording electrode, a glass capillary GC100-10 (Harvard Apparatus, Holliston, MA, USA) with a tip diameter of 20–30 μm filled with E3, was placed on top of the cornea.

#### 2.7.1. White light electroretinography

For *eaat5b* mutant and the corresponding WT control recordings, ERG setup 1 with light source 1 (ZEISS XBO 75 W) and interface (NI PCI-6035E through NI BNC-2090 accessories, National Instruments) described previously was used [35]. For *eaat7* mutants, *eaat5b/eaat7* double mutant and the corresponding WT control recordings, ERG setup 2 with light source 2 (HPX-2000 xenon light source, Ocean Optics) and interface (SCC-68, National Instruments) described previously was used [36]. The spectra of both ERG light sources were measured by a spectrometer (Ocean Optics, USB2000b; software Spectra Suite, Ocean Optics; electronic supplementary material, figure S1). The light intensity is calculated as the area under the curve. The light intensity of light source 1 is 1874.4 μW cm^−2^ and the light intensity of light source 2 is 12 652.8 μW cm^−2^ between 189 and 800 nm. A series of five white light stimuli of increasing light intensities (log −4 to log 0) were presented to the eyes. The duration of stimuli was 100 ms with inter-stimulus intervals of 7 s.

The ERG traces were analysed using Excel and Igor-Pro software (Wave Metrics). The *b*-wave amplitudes were analysed as a proxy for ON-bipolar cell depolarization. The first 50 ms of each recording were averaged and taken as baseline values. The *b*-wave amplitudes were statistically compared between WT and mutant animals by a two-tailed *t*‐test using IBM SPSS Statistics v. 22.

The *b*-wave implicit time was analysed as a read-out for transporter kinetics. The implicit time is defined as the time from light onset to the onset of the *b*-wave. By extrapolating a line that goes through two points on the curve at *Y* = 80% and *Y* = 20% of the *b*-wave amplitude, respectively, the implicit time was defined as the intersection of this line and the *X*-axis. Values below 50 and above 230 ms were filtered out. Implicit times were statistically compared between WT and mutants with a two-tailed *t*‐test using IBM SPSS Statistics v. 22.

All ERG results are plotted in box-and-whisker plots with boxes reaching from the first to the third quartile, the line within the box representing the median and top and bottom whiskers reaching to the maximum or minimum of obtained values, respectively.

Example single traces are depicted in electronic supplementary material, figure S2.

#### 2.7.2. Flicker electroretinography

The flicker fusion frequency (FFF) defined as the frequency of visual stimuli that can no longer be temporally resolved was assessed as a read-out for temporal resolution of vision. A total of 15 ms light stimuli were presented to the eyes at different frequencies starting at 7 Hz and increasing in 1 Hz steps. ERG responses to each frequency were recorded for 2 s.

The power of each response within 0 to 50 Hz range was extracted using the fast Fourier transform algorithm in MATLAB (Supplementary code). A peak at a given frequency indicates that the frequency can be temporally resolved; absence of a peak indicates it cannot be resolved. The highest resolvable frequency, referred to as the FFF, was determined. A two-tailed *t*‐test was conducted using IBM SPSS Statistics v. 22 to statistically compare the FFFs between mutant and WT animals.

### 2.8. Image processing and assembly

Images were selected and processed, including fine-tuning of contrast and colour balance and assembling *z*-stacks, using Imaris (Bitplane) and Adobe Photoshop CC 2017 and assembled using Adobe Illustrator CC 2017.

### 2.9. Quantitative reverse transcription PCR

The 5 dpf larvae were anaesthetized on ice and eyes were dissected using an insect pin and a syringe needle in a dish containing RNA later (Sigma-Aldrich). To confirm genotype, remaining tissue was lysed and gDNA was amplified by means of PCR (KAPA2G Fast HotStart PCR kit, KAPA Biosystems) as described above. The total RNA of pools of larval eyes (18–28 per sample) was extracted using the RNeasy Plus Mini kit (Qiagen). RNA was reverse transcribed to cDNA using the Super Script III First-strand synthesis system (Invitrogen) using 1:1 ratio of random hexamers and oligo (dt) primers.

qPCR reactions were performed using SsoAdvanced Universal SYBR Green Supermix on a CFX96 Touch Real-Time PCR Detection System (Bio-Rad). Primer efficiencies were calculated by carrying out a dilution series. After the primer efficiencies were determined equal, eye samples were used for qPCR using 1 ng of cDNA per reaction. The controls ‘no reverse transcription control’ (nRT control) and ‘no template control’ (NTC) were performed with every qPCR reaction. The *rpl13a* was chosen as a reference gene. All reactions were performed in technical triplicates. Data were analysed in CFX Maestro software from Bio-Rad, Microsoft Excel and R. Statistical analysis was performed on the log-transformed normalized expression per sample.

Primers used were *eaat7* (GCCCACTCACGACAACCAG; ATCTCGTTCCCGTTCCCTCAG; amplicon size 133 bp), *eaat5b* (GCTGATGCGCATGTTGAAGATG; GACGACTGGAACACTTGGCATC; 95 bp) and *rpl13a* (TCTGGAGGACTGTAAGAGGTATGC; AGACGCACAATCTTGAGAGCAG; 148 bp).

### 2.10. Heterologous expression of EAAT fusion proteins in mammalian cells

Transient transfection of HEK293T (Invitrogen) with the EAAT–YFP fusion proteins *dr*EAAT7, *r*EAAT4 and *h*EAAT1 in pcDNA3.1 vectors (Invitrogen) was performed using the Ca^2+^ phosphate technique as previously described [37].

### 2.11. Confocal microscopy of EAAT7 fusion proteins

Confocal imaging was carried out on living cells grown on poly-lysine coated *µ*-dishes (ibidi) without fixation using an inverted confocal laser scanning microscope (Leica TCS SP5; Leica Microsystems, Heidelberg, Germany) equipped with a 63×/1.4 oil immersion objective. YFP was excited using a 488 nm laser and emitted light was recorded between 525 and 540 nm. Spatial distributions of YFP fluorescence were analysed with Fiji package (NIH).

### 2.12. Electrophysiology

Standard whole-cell patch clamp recordings were performed using an Axoclamp 200B (Molecular Devices) amplifier. Borosilicate glass electrodes were pulled and used with resistances between 1 and 2.5 MΩ. Compensation of 80% of the series resistance reduced voltage errors. Recorded currents were filtered at 1 kHz and digitized by a Digidata 1550A (Molecular Devices) digitizer at a sampling rate of 10 kHz. To record anion currents, currents were measured using standard external solution containing in the bath (mM): 140 NaNO_3_, 4 KCl, 2 CaCl_2_, 1 MgCl_2_, 0.1 l-glutamate and 10 HEPES/NaOH, pH 7.4, and in the pipette: 110 KNO_3_ or NaNO_3_, 2 MgCl_2_, 5 EGTA and 10 HEPES (KOH or NaOH), pH 7.4. For measurements of glutamate transport, all anions were equimolar replaced by the impermeable corresponding gluconate salt. All experiments were carried out using agar bridges with 2 M KCl in 1% agarose to connect the Ag/AgCl electrode. Liquid junction potential differences were calculated in pClamp 10.5 (Molecular Devices) and protocols were adjusted *a priori*. Currents were analysed using Clampfit (Molecular Devices) and Sigma Plot (SysStat) software. Current–voltage relationships show point plots of mean current amplitudes with standard errors. Box plots of current amplitudes at a single holding potential span the first to the third quartiles, with whiskers reaching maximum and minimum values, respectively. The lines indicate the median values. Pair-wise comparison of datasets was done with one-way ANOVA and Holm–Sidak *post hoc* testing.

## 3. Results

### 3.1. *EAAT5b* and *EAAT7* are expressed on ON-bipolar cell dendritic tips

In order to identify EAATs that are potentially involved in synaptic transmission at the photoreceptor synapse, we cloned all 11 orthologues in zebrafish and performed an mRNA *in situ* expression survey for expression in second-order neurons in the retina (M. Gesemann, unpublished data). For two orthologues, *eaat5b* and *eaat7*, we found expression in the inner nuclear layer (INL) of the retina making them candidates as mediators in direct synaptic transmission.

**Figure 1.**
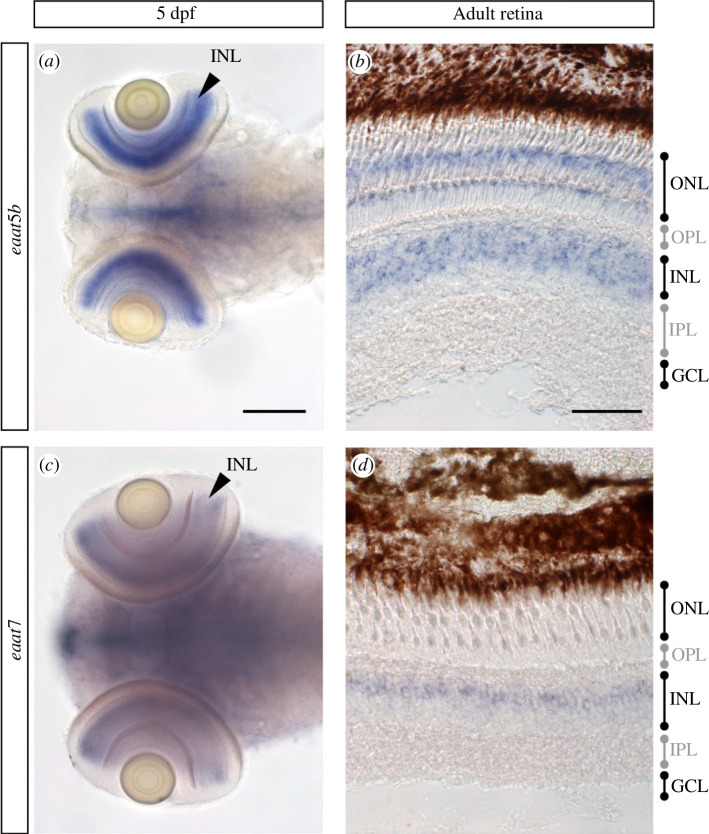
*eaat5b* and *eaat7* mRNA is detected in the INL of the retina. (*a,b*) The transcripts of *eaat5b* are found in the INL at both larval and adult stages. (*b*) In the adult retina, *eaat5b* mRNA is further detected in the ONL. (*c*) *eaat7* transcripts in the retina are exclusively found in the INL in larvae as well as in the adult eye (*d*). Scale bar is 100 μm in (*a,c*) and 50 μm in (*b,d*). PhR, photoreceptor; OPL, outer plexiform layer; INL, inner nuclear layer; ONL, outer nuclear layer; IPL, inner plexiform layer; GCL, ganglion cell layer.

In the larval retina, the transcript of *eaat5b* is exclusively found in the INL (figure 1*a*). During adult stages, *eaat5b* mRNA is additionally expressed in the outer nuclear layer (ONL) (figure 1*b*). The *eaat7* message is found in cells of the INL, both in 5 dpf larvae and in the adult retina (figure 1*c,d*). Furthermore, both *eaat5b* and *eaat7* mRNA are additionally detected in other regions of the central nervous system, which however is not the subject of this study.

**Figure 2.**
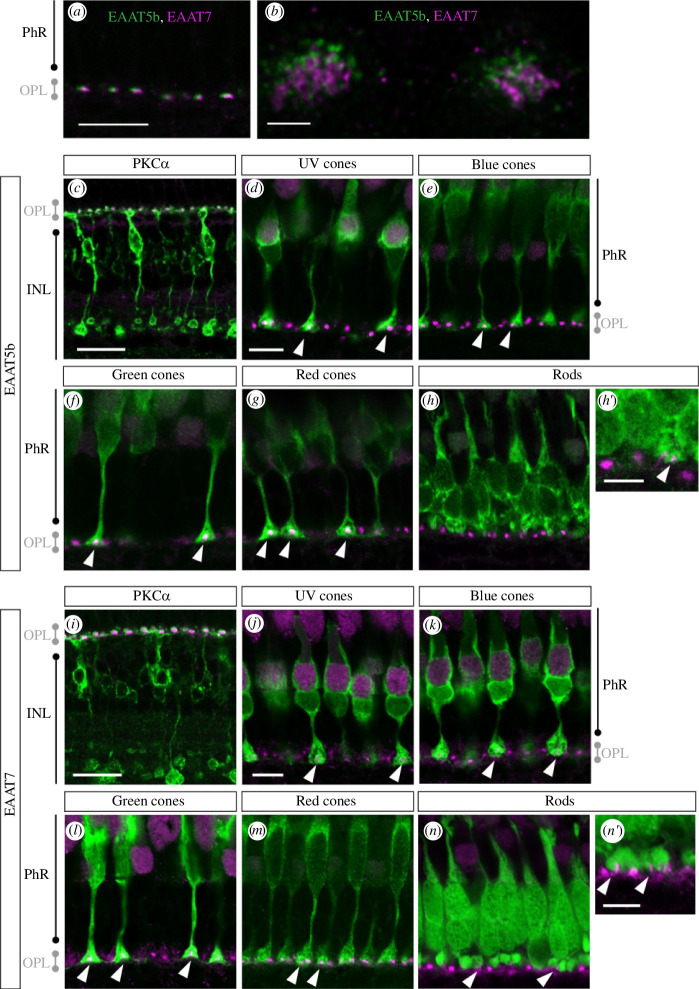
EAAT5b and EAAT7 co-localize on ON-bipolar cell dendritic tips. (*a,b*) Adult retinal sections stained with anti-EAAT5b (green) and anti-EAAT7 antibodies (magenta) show punctate co-localization of EAAT5b and EAAT7 in the OPL. (*c*) Immunostaining of EAAT5b (magenta) and PKCα (green) demonstrates dendritic localization of EAAT5b on ON-bipolar cells. EAAT5b localizes to ON-bipolar cells contacting UV (*d*), blue (*e*), green (*f*) and red (*g*) cones but only a minority of rods (*h,h′*). Similarly, EAAT7 (magenta) is specific to ON-bipolar cell (stained with PKCα in green) dendrites (*i*) and localizes to UV (*j*), blue (*k*), green (*I*) and red (*m*) cone- and few rod synapses (*n,n′*). Scale bar in (*a*) is 15 µm, scale bar in (*b*) is 1 µm, scale bar in (*c*) is 20 µm, scale bar in (*d*) is 8 µm, scale bar in (*h′*) is 5 µm, scale bar in (*l*) is 20 µm, scale bar in (*j–n*) is 8 µm, scale bar in (*n′*) is 5 µm. PhR, photoreceptor; OPL, outer plexiform layer; INL, inner nuclear layer.

To assign the expression to specific inner retinal cell types and to access subcellular localization, we generated paralogue-specific polyclonal antibodies. These antibodies were used for immunohistochemical staining. Both transporters share a common dotted expression pattern in the outer plexiform layer (OPL). Co-staining of EAAT5b and EAAT7 with the ON-bipolar cell marker PKCα [38] reveals that both transporters localize to dendritic tips of ON-bipolar cells (figure 2*c,i*). EAAT5b and EAAT7 are always co-expressed within a dendritic tip (figure 2*a,b*). Surprisingly, super-resolution microscopy reveals adjacent but not overlapping localization within a dendritic terminal (figure 2*b*). While EAAT5b localizes more proximal to the synaptic cleft, EAAT7 is located slightly more distal to it. Next, we asked which photoreceptor subtypes are contacting the EAAT5b/7 positive synapses. Therefore, we used a number of transgenic lines expressing GFP under the control of different opsin promoters, namely *Tg(zfSWS1−5.5A:EGFP)* [25], *Tg(zfSWS2−3.5A:EGFP)* [26], *Tg(zfRh2−2:EGFP)* [27], *Tg(zfLWS:EGFP)* [28] and *Tg(zfRh1−3:EGFP)* [29]. Immunolabelling on retinal sections of these transgenic lines shows that EAAT5b as well as EAAT7 proteins localize to ON-bipolar cell dendrites contacting all four cone subtypes (UV-, blue-, green- and red-light-sensitive cones) but only a minority of rod spherules (figure 2*d–h′* and *j–n′*). Around 21.7 ± 3.3% (mean ± s.e.m., *n* = 3) of rod synapses contact EAAT5b positive cells and about 25.5 ± 3.0% (mean ± s.e.m., *n* = 5) of rod synapses contact EAAT7 positive cells. Taken together, these data imply that these two transporters participate in cone signal transmission.

### 3.2. Loss of EAAT5b and EAAT7 does not affect retinal morphology and synaptic structure

In an effort to elucidate the function of these transports/receptors, we generated KO mutant strains using CRISPR/Cas9 genome editing technology. Both generated genome modifications are predicted to result in severely truncated proteins of no function (electronic supplementary material, figure S3). As both antibody binding epitopes are either C-terminal of the predicted stop codon (EAAT5b) or disrupted by the deletion (EAAT7) (electronic supplementary material, figure S3), neither EAAT5b nor EAAT7 was detected in the corresponding mutants. This provides additional evidence for the paralogue specificity of the generated antibodies.

**Figure 3.**
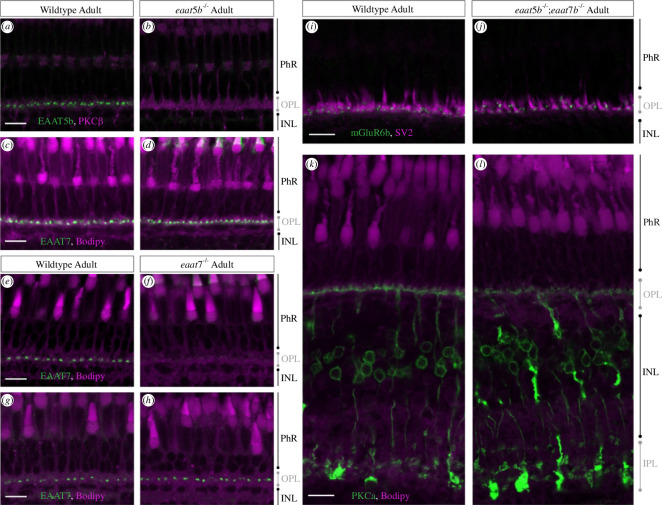
Normal retinal morphology in *eaat5b*−/−, *eaat7*−/− and double KO animals. Immunofluorescent staining of EAAT5b (green) on WT (*a*) and *eaat5b* mutant retina (*b*) reveals the absence of EAAT5b protein in mutants. Mutation in *eaat5b* does not affect localization and abundance of EAAT7 (green), seen by comparable immunofluorescence in WT (*c*) and *eaat5b* mutants (*d*). EAAT7 antibody staining on WT (*e*) and *eaat7* KO retina (*f*) reveals no EAAT7 protein (green) immunofluorescence can be detected in *eaat7*−/− retina (*f*); however, the expression pattern of EAAT5b in *eaat7* KO animals (*h*) is not affected (if compared with WT (*g*)). Synaptic structure as well as ON-bipolar cell morphology in double KO animals are not affected. Both the localization of pre-synaptic marker SV2 (magenta) as well as the post-synaptic mGluR6b (green) are not altered in the retina of *eaat5b*−/−;*eaat7*−/− double mutants (*j*) if compared with WT (*i*). Furthermore, shape of ON-bipolar cells stained with PKCα (green) is comparable between double mutants (*l*) and WT (*k*). Scale bars in (*a*,*c*,*e*,*g*,*l*,*k*) are all 10 μm and also correspond to (*b*,*d*,*f*,*h*,*j*,*l*), respectively.

Homozygous mutation of *eaat5b* does not result in any obvious alterations in staining abundance and localization of EAAT7 (figure 3*b*) and, vice versa, *eaat7* KO animals show an undisrupted distribution of EAAT5b (figure 3*d*). In order to provide a more quantifiable measure, we performed quantitative PCR on both mutant strains and indeed found no changes in *eaat5b* mRNA abundance in *eaat7*−/− mutants and vice versa of *eaat7* mRNA in *eaat5b*−/− mutant larvae (electronic supplementary material, figure S4). The combined loss of both transporters, EAAT5b and EAAT7, has no influence on synaptic structure nor localization of synaptic proteins such as the pre-synaptic marker SV2 or the post-synaptically expressed metabotropic glutamate receptor 6b (mGluR6b) (figure 3*i–j′*). Additionally, the morphology of ON-bipolar cells visualized by PKCα immunofluorescence appears normal in double mutants (figure 3*k–l′*).

Retinal morphology of 5 dpf larvae and adult eyes of the homozygous mutant was assessed on histological plastic sections. Loss of either EAAT5b or EAAT7 does not cause any retinal morphology changes. Lamination as well as thickness of the retinal layers appear normal in the KO animals (electronic supplementary material, figure S5*c*–*f*). Furthermore, also double KO animals (*eaat5b−/−*; *eaat7−/−*) did not reveal any morphological alterations of the retina, neither at larval nor at adult stages (electronic supplementary material, figure S5*g*,*h*).

In summary, neither retinal morphology alterations nor changes in synaptic protein distribution and abundance are apparent in single and double mutants, indicating that any functional phenotype does not arise from structural changes but could be solely attributable to functional alterations of the corresponding transporter.

### 3.3. EAAT5b and EAAT7 contribute to the ON-response

**Figure 4.**
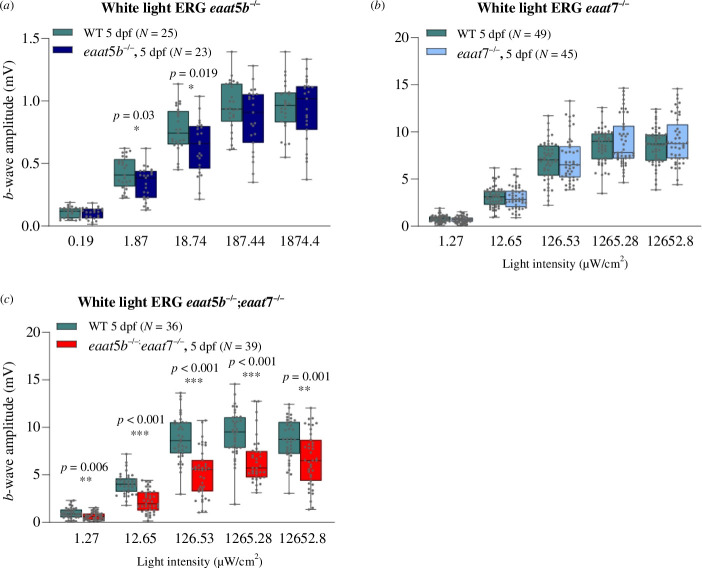
Involvement of EAAT5b and EAAT7 in ON-bipolar cell activation. ERG was recorded on *eaat5b−/−* (*a*), *eaat7−/−* (*b*) and *eaat5b−/−*;*eaat7−/−* (*c*) animals. The *b*-wave amplitudes are plotted in box-and-whisker plots. (*a*) Loss of EAAT5b causes a small, slightly significant decrease in the *b*-wave amplitude for medium light intensities (log –3 and log –2). (*b*) Homozygous mutation in *eaat7* does not affect ON-bipolar cell activation in (*a*) by white light ERG measurable way. (*c*) Double KO animals show a significant reduction in the *b*-wave amplitudes across the five irradiances. Note that the data plotted in (*a*) were recorded at lower light intensities than in (*b,c*).

Next, we assessed outer retinal function in the mutants. We employed ERG, providing a ready assessment of outer retinal function. We quantified the *b*-wave in larval retinae as a read-out of ON-bipolar cell depolarization. While the loss of EAAT5b causes a subtle decrease in the *b*-wave amplitude at medium light intensities (log –3 and log –2 of the maximum light intensity of 1874.4 μW cm^−2^), the *b*-wave amplitudes for dim light (log –4) and bright light (log –1 and log 0) do not significantly differ between WT and *eaat5b* KO animals (figure 4*a*). Homozygous mutant larvae for *eaat7* show no phenotype in the ERG upon white light stimulation at all light intensities tested between around 1.3 and 12 652 μW cm^−2^ (figure 4*b*).

However, double KO larvae lacking both EAAT5b and EAAT7 reveal a strong ERG phenotype. The *b*-wave amplitudes of *eaat5b−/−*;*eaat7−/−* are strongly reduced across all light intensities tested between around 1.3 and 12 652.8 μW cm^−2^, indicating a redundant involvement of these two transporters in ON-bipolar cell activation (figure 4*c*).

**Figure 5.**
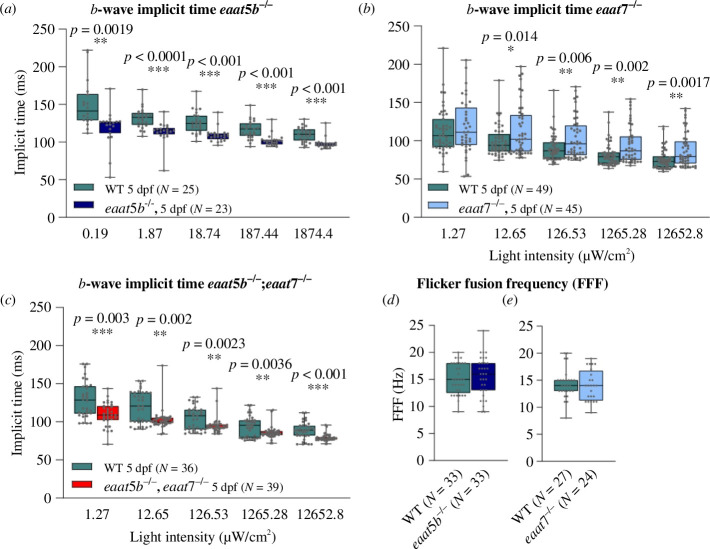
EAAT5b and EAAT7 differentially shape the ON-response but do not influence the FFF. The time from light onset to the initiation of the *b*-wave was quantified and plotted in box-and-whisker plots for *eaat5b* mutant (*a*), *eaat7* mutant (*b*) and double KO animals (*c*). Loss of EAAT5b significantly reduces the implicit time across all irradiances (*a*). Mutation in *eaat7* results in an increased implicit time (*b*). If both transporters are lost, the ON-response implicit time is decreased (*c*), as in the *eaat5b* single mutant. Nevertheless, the slow gating mechanism of EAAT5b is not affecting the temporal resolution of vision, as both *eaat5b* (*d*) as well as *eaat7* mutant (*e*) animals display an FFF similar to WT.

We next investigated the kinetics of signal transmission in these mutants. Interestingly, the loss of the two ON-bipolar cell glutamate transporters differentially affects the kinetics of signal transmission in the first visual synapse. The *b*-wave implicit time, defined as the time from the onset of the light stimulus to the onset of the *b*-wave, is significantly decreased in *eaat5b* mutant animals compared to WT (figure 5*a*). This demonstrates that the ON-response commences faster in animals without a functional EAAT5b transporter throughout all light intensities tested between around 0.19 and 1874.4 μW cm^−2^, indicating slow gating mechanisms of EAAT5b.

Conversely, the ON-response initiation in *eaat7* mutants is delayed compared with WT animals for all light intensities except for very dim light stimuli around 1.3 μW cm^−2^ (figure 5*b*), indicative for different gating kinetics between EAAT5b and EAAT7. The *eaat5b;eaat7* double KO resembles the single *eaat5b* mutant in showing a decrease in the ON-response implicit time for all light intensities between around 1.3 and 12 652.8 μW cm^−2^ (figure 5*c*).

In order to further explore the kinetic properties of the mutants, we assessed the FFF by ERG. This method measures the time for full recovery of the ERG response of a light flash after a conditioning flash of light of the same light intensity. Despite the differences in gating kinetics, no significant change in the FFF could be observed either in *eaat5b−/−* or in *eaat7−/−* (figure 5*d,e*).

### 3.4. Electrophysiological characterization of *dr*EAAT7 in HEK293T cells

**Figure 6.**
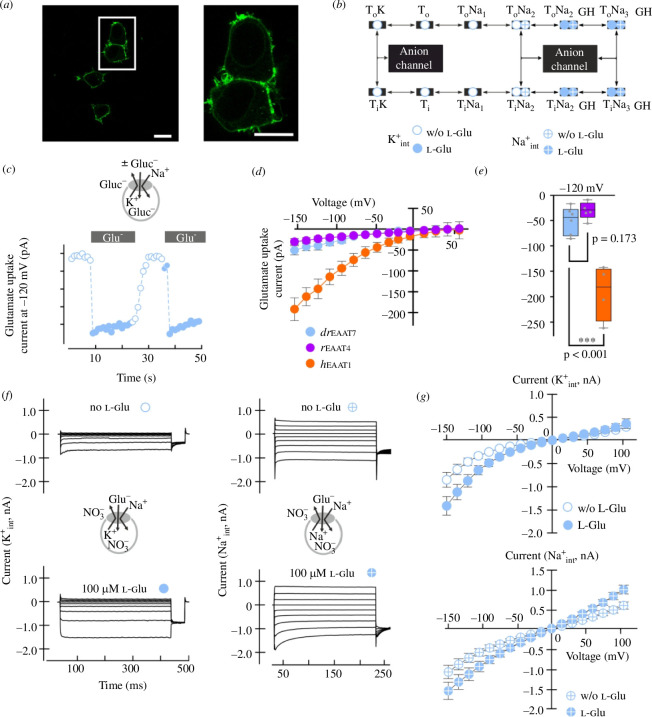
Zebrafish EAAT7 exhibits low transport capability and a dominating anion conductance. (*a*) Confocal microscopy indicates an almost exclusive insertion of the EAAT7–YFP fusion protein into the cell membrane (scale bars: 10 µm). (*b*) Scheme of the full transport cycle (open circles) and the Na^+^ exchange half-cycle (crossed circles) depict the transitions from transport mode to anion channel openings during the intermediate conformations between outward (o) and inward (i) facing conformations. (*c*) Whole-cell glutamate transport currents in the absence of permeable anions at a holding potential of −120 mV over time. The applications of 5 mM l-glutamate are indicated by grey bars (controls: open circles, with glutamate: filled circles). (*d*) Current–voltage relationships of transport currents for *dr*EAAT7, *r*EAAT4 and *h*EAAT1, under ionic conditions as used in (*c*) (*n* = 6/5/4). (*e*) Boxplot of transport current amplitudes at −120 mV for *dr*EAAT7, *r*EAAT4 and the high-capacity transporter *h*EAAT1. (*g*) Representative whole-cell currents in the presence of the permeable anion NO_3_^−^ under control conditions (top) and in the presence of 0.1 mM l-glutamate in the bath. Currents are shown for ionic conditions permitting the full transport cycle (K^+^_int_, left), and restricting the transporter conformations to the exchange half-cycle (Na^+^_int_, right). (*h*) Current–voltage relationships for the whole-cell anion currents shown in (*g*) for both conditions (*n* = 7/7).

We next expressed *dr*EAAT7 and *dr*EAAT5b fused to YFP transiently in HEK293T cells and tested the surface insertion of *dr*EAAT7-YFP with confocal microscopy. Confocal images and fluorescence intensity plots of cell passing transects showed almost exclusive insertion of WT *dr*EAAT7–YFP into the surface membrane, or in domains in close proximity (figure 6*a*). EAAT5b was only expressed at low levels, and we could not detect clear membrane staining (electronic supplementary material, figure S6); therefore no electrophysiological characterization of *dr*EAAT5b was possible in mammalian cells. The EAATs are secondary-active co-transporters for glutamate and anion channels [16,39–41], and we therefore had to experimentally separate the two transport functions of *dr*EAAT7. Glutamate is co-transported into cells together with 3 Na^+^ and 1 H^+^, followed by the K^+^-bound re-translocation of the transporter back to the outward-facing state and anion channel openings occur from intermediate conformations (figure 6*b*). We tested *dr*EAAT7-mediated glutamate transport by whole-cell patch clamping under conditions abolishing EAAT anion currents: cells were internally dialysed with K^+^ gluconate and externally perfused with Na^+^ gluconate [42,43]. Cells were clamped at a holding potential of −120 mV, and glutamate uptake was evoked by adding 5 mM to the external solution. Glutamate caused a reversible inward current in cells expressing zebrafish EAAT7 (figure 6*c*), but not in untransfected control cells (data not shown). Figure 6*d*,*e* compares uptake currents mediated by zebrafish EAAT7 to human EAAT1 and rat EAAT4 uptake currents (*n* = 6/5/4). The *h*EAAT1 represents an efficient glial glutamate transporter [42,44,45], and *r*EAAT4 is a prototypical glutamate transporter with low transport capability and high anion conductance [46–48]. The uptake capability of zebrafish EAAT7 is similar to that of *r*EAAT4 (*p* = 0.332), but much smaller than that of *h*EAAT1 (*p* < 0.001; figure 6*d,e*).

EAATs transport either Na^+^ together with glutamate and H^+^ or K^+^ in two half-cycles, often denoted as Na^+^ and K^+^ hemicycles (figure 6*b*). To record anion currents associated with both hemicycles [39], we dialysed cells with NO_3_^−^-based solutions supplemented with K^+^. Anion channel activity associated with the Na^+^-hemicycle was measured with Na^+^ as the main internal cation (figure 6*g,h*). Application of glutamate increased currents at negative holding potentials under uptake conditions (figure 6*h*, −150 mV , *p* = 0.016 (K^+^_int_, *n* = 7)), but not under exchange conditions (*p* = 0.107 (Na^+^_int_, *n* = 7)). In the presence of K^+^_int_, this increase was observed only at negative voltages, while during depolarization the currents were equal with or without glutamate in the bath (+75 mV, *p* = 0.515). With internal sodium (Na^+^_int_) significant current increases were observed only at positive holding potentials (+75 mV, *p* = 0.017). We conclude that *dr*EAAT7 is a functional glutamate transporter with a dominating anion conductance.

## 4. Discussion

In darkness, photoreceptors tonically release the excitatory neurotransmitter glutamate. Upon light stimulation, photoreceptors hyperpolarize leading to a reduction or cessation of glutamate release. ON-bipolar cells react to the decreasing glutamate concentration in the synaptic cleft by depolarization. This paradoxical inhibition by the quintessential excitatory neurotransmitter glutamate is thought to be mediated by the G-protein coupled mGluR6–TRPM1 pathway and is conserved across vertebrates [2,4,49]. A number of studies conducted on different vertebrates suggest an additional non-metabotropic glutamatergic mechanism to be involved in ON-bipolar cell activation, mainly in teleosts [6,50] but also in mammals [51]. The described glutamate-gated chloride current *I*_Glu_ hyperpolarizes ON-bipolar cells in darkness, in the presence of glutamate. Decreasing glutamate concentrations in the synaptic cleft upon light activation reduced this current and caused depolarization of ON-bipolar cells. The *I*_Glu_ was shown to be sensitive to the non-specific EAAT blocker TBOA, implying it to be generated by a glutamate transporter associated with high Cl^−^ conductance [7].

It is still not clear as to which EAAT family members are responsible for glutamate reuptake in the retina and the details of the interplay between EAATs with the well-defined mGluR6 pathway remain equally unresolved. Previous research proposes a role for EAATs in cone signalling, while mGluR6 mainly functions in rod signalling in the giant danio [9]. Other studies suggest a spectral difference between mGluR6 and EAAT signalling. mGluR signalling is thought to mainly mediate short-wavelength photopic signalling, while calculations of the spectral sensitivity of the non-metabotropic signalling revealed it to be distributed across the visible spectrum [52,53].

In the present study, we identified EAAT5b and EAAT7 as the glutamate transporters that mediate direct synaptic transmission in the outer retina. EAAT5b and EAAT7 were found to be present on dendritic tips of ON-bipolar cells and therefore in the expected localization for mediating the hyperpolarizing Cl^−^ current in the presence of glutamate, described by others [6–8,50,53]. Importantly, biophysical measurements confirmed that EAAT7 is indeed a functional glutamate transporter with a predominant anion conductance. EAAT5b and EAAT7 co-localize, while not overlapping, on ON-bipolar cell dendritic terminals that are in contact with all four cone subtypes but only a limited number of rod spherules, indicating an important function in cone signal transmission. Expression of EAAT5b and EAAT7 does not indicate a spectral difference in the involvement of glutamatergic signalling pathways activating ON-bipolar cells as localization does not discriminate between synapses of different cone types.

In order to assess the function of these two EAATs in synaptic transmission, we generated single-KO animals and double mutants. No changes in retinal morphology and localization of various synaptic markers were apparent in these mutants. Functional analysis of the two glutamate transporters in the retina was performed by ERG recordings, focusing on the *b*-wave as a read-out of ON-bipolar cell depolarization. It is important to note that at the developmental stages used (5 dpf), rods do not significantly contribute to the ERG, hence the measured responses reflect pure cone responses [54,55]. The lack of EAAT5b causes a slight decrease in the *b*-wave amplitude for medium light intensities, indicating that EAAT5b contributes to the hyperpolarization of ON-bipolar cells in darkness, albeit to a small extent. The *eaat5b/eaat7* double mutants showed a robust reduction of the *b*-wave, proving a redundant involvement of these two glutamate transporter in the ON-response of the cone pathway. The remaining *b*-wave in the double mutant argues for a joined contribution of non-metabotropic (EAAT) and metabotropic (mGluR6) signalling in the generation of the photopic ON-response. Interestingly, studies in adult giant danio, a closely related species, presented evidence for a much larger contribution of EAATs on the cone ON-response [9,10]. In a recent study, we found only a small residual *b*-wave activity in mGluR6b KO fish (Haug *et al*., unpublished).

The *eaat5b* KO animals show a significant decrease in the ON-response implicit time, meaning that initiation of ON-bipolar cell depolarization is accelerated in mutants. This observation precludes the possibility of EAAT5b being a high-capacity transporter, as a lack of such a high-capacity transporter would cause a delay of the ON-response caused by remaining cleft glutamate even upon a light stimulus. The analysis of the ERG components by Nelson & Singla [53] revealed that the non-metabotropic ON-response consists of a short negative deflecting wave followed by the bigger positive deflecting *b*-wave. This post-photoreceptor *a*-wave is probably caused by the Cl^−^ current that is reduced when glutamate levels decrease at light on [53]. The mammalian retina-specific EAAT5 has been associated with slow gating mechanisms [21], and it is therefore tempting to speculate that zebrafish EAAT5b also exhibits slow activation and deactivation and that the post-photoreceptor *a*-wave is generated by such slowly activating and deactivating Cl^−^ currents. The loss of EAAT5b would cause the abolishment of this post-photoreceptor *a*-wave allowing ON-bipolar cells to depolarize faster, as seen in *eaat5b* mutant animals. Remarkably, EAAT5 is pre-synaptically expressed in the mouse retina and is the source of autoinhibition of rod bipolar cells [22,56].

EAAT7, albeit absent in mammalian retinas, is associated with fast kinetics. *eaat5b*−/−;*eaat7−/−* double knockout animals displayed the same acceleration in the ON-bipolar cell depolarization as *eaat5b* single mutants, supporting the hypothesis of the slow initiation of the ON-response being caused by the short hyperpolarization of ON-bipolar cells at light on (post-photoreceptor *a*-wave). The observed kinetics of the ON-response are especially remarkable considering that ON-bipolar cell activation in double mutants is solely mediated by metabotropic signalling, which is generally assumed to be slow owing to the involvement of second messengers (G-protein signalling). Nevertheless, our kinetic analysis demonstrates that mGluR6-mediated depolarization of ON-bipolar cells is of a faster time frame than EAAT5b-mediated ON-bipolar cell depolarization. The slow kinetics of EAAT5b has no influence on temporal resolution of vision, as the FFF in *eaat5b* and *eaat7* KO animals is comparable to the one in WT animals, indicating that other mechanisms are the rate-limiting factors of FFF. Interestingly, in the mouse retina (which lacks EAAT7) EAAT5 protein was found to improve the temporal resolution through a pre-synaptic mechanism in bipolar cells [22].

Taken together, EAAT5b and EAAT7 are the two transporters that mediate the previously described glutamate-gated Cl^−^ current in zebrafish ON-bipolar cells. Thus, in darkness when glutamate is maximally released, the Cl^−^ flux through EAATs renders ON-bipolar cells hyperpolarized. At light on, termination of glutamate release by photoreceptors causes ON-bipolar cells to depolarize owing to the termination of the EAAT-mediated Cl^−^ current and the opening of the cation conducting TRPM1 channel mediated through mGluR6 signalling. We showed that EAAT5b and EAAT7 are post-synaptically present in ON-bipolar cell synapses contacted by all cone subtypes. Loss of these EAATs leads to a diminished ON-response, proving their partially redundant involvement in direct synaptic transmission. Having a dual glutamatergic input system harbours several advantages. Rods seem to mainly rely on metabotropic signalling which allows signal amplification. Furthermore, EAATs and mGluRs are thought to have different glutamate dose response functions. The demonstrated kinetic effect on the ON-response allows for an additional level of temporal regulations. Salamander EAAT5 expressed in *Xenopus* oocytes was shown to have a glutamate dose response over 4 log units [57], while mGluR6 glutamate dose response saturates at 2 log units [58]. And last but not least, mGluRs are not only involved in signal transmission but are key molecules in mediating the proper localization of synaptic proteins [59,60].

## Data Availability

Raw data will be provided on request to the lead author. Supplementary material is available online [61].
